# Quantifying the Impact of Deprivation on Preterm Births: A Retrospective Cohort Study

**DOI:** 10.1371/journal.pone.0023163

**Published:** 2011-08-03

**Authors:** David Taylor-Robinson, Umber Agarwal, Peter J. Diggle, Mary Jane Platt, Bill Yoxall, Zarko Alfirevic

**Affiliations:** 1 Department of Public Health and Policy, University of Liverpool, Liverpool, United Kingdom; 2 Liverpool Women's National Health Service Foundation Trust, Liverpool, United Kingdom; 3 School of Health & Medicine, Lancaster University, Lancaster, United Kingdom; 4 Health Policy and Practice, University of East Anglia, Norwich, United Kingdom; 5 Department for Women's and Children's Health, University of Liverpool, Liverpool, United Kingdom; The University of Adelaide, Australia

## Abstract

**Background:**

Social deprivation is associated with higher rates of preterm birth and subsequent infant mortality. Our objective was to identify risk factors for preterm birth in the UK's largest maternity unit, with a particular focus on social deprivation, and related factors.

**Methodology/Principal Findings:**

Retrospective cohort study of 39,873 women in Liverpool, UK, from 2002–2008. Singleton pregnancies were stratified into uncomplicated low risk pregnancies and a high risk group complicated by medical problems. Multiple logistic regression, and generalized additive models were used to explore the effect of covariates including area deprivation, smoking status, BMI, parity and ethnicity on the risk of preterm birth (34^+0^ weeks). In the low risk group, preterm birth rates increased with deprivation, reaching 1.6% (CI_95_ 1.4 to 1.8) in the most deprived quintile; the unadjusted odds ratio comparing an individual in the most deprived quintile, to one in the least deprived quintile was 1.5 (CI_95_ 1.2 to 1.9). Being underweight and smoking were both independently associated with preterm birth in the low risk group, and adjusting for these factors explained the association between deprivation and preterm birth. Preterm birth was five times more likely in the high risk group (RR 4.8 CI_95_ 4.3 to 5.4), and there was no significant relationship with deprivation.

**Conclusions:**

Deprivation has significant impact on preterm birth rates in low risk women. The relationship between low socio-economic status and preterm births appears to be related to low maternal weight and smoking in more deprived groups.

## Introduction

Preterm birth is an important public health issue in the UK and internationally, since prematurity is a major contributor to infant mortality and inequalities in health [1], [2], [3], [4]. Preterm birth rates are rising in countries such as the UK, the US and the Scandinavian countries over the past 20 years, under the influence of factors such as assisted conception and increasing maternal age in pregnancy [3]. The aetiology of preterm birth is complex, and previous studies have identified a plethora of individual and environmental level factors as being important in the pathway to preterm births [5], [6], [7]. A number of recurring socio-demographic, obstetric and medical risk factors have been identified, including socio-economic status (SES) [7], [8], [9], [10], [11], [12] ENREF6. Understanding the relationship of these factors is central to designing effective prevention strategies [4], [13].

There is increasing interest in the UK in publishing and comparing clinical outcomes across centres [14], but these comparisons are likely to be confounded by socioeconomic and other case-mix factors. An objective in this study was to better understand the influence of SES on risk of preterm birth in one of the most socially deprived urban areas in the UK, and to explore the effect of SES on both low and high risk populations. A recent study by Smith et al. concluded that almost 80% of the relative deprivation gap in all cause neonatal mortality is due to premature birth and congenital anomalies and stated that “Understanding the link between deprivation and preterm birth should be a major research priority to identify interventions to reduce preterm birth” [4]. It has been hypothesised that the mechanism linking low SES to preterm birth may be explained by the clustering of individual level risk factors in women from more disadvantaged backgrounds [6], [8]. In order to explore the relationship between SES and preterm birth it is necessary to account for these factors in the analysis. For instance, in another recent study Smith et al demonstrated higher rates of very preterm birth across deprivation quintiles in the Trent region, but they were unable to explore aetiological factors such as cigarette smoking, ethnicity, and history of previous preterm birth [15].

Since the aetiology of preterm birth is significantly different in multiple pregnancies, women with a previous history of preterm birth, and following intrauterine transfers to tertiary obstetric units, we aimed to explore the effect of SES in singleton, booked pregnancies regarded clinically as either high or low risk. Our a priori hypothesis was that there would be differential rates of preterm birth by socioeconomic status, and that these might be related to individual level risk factors. We show that deprivation is indeed an important risk factor in low risk pregnancies, and that this is related to maternal smoking and underweight.

## Methods

### Ethics Statement

Ethical approval for this study was sought and granted by the Sefton Research Ethics Committee. We used routinely collected data from electronic hospital records, analysed anonymously, and individual patient consent was not required.

### Objective

To quantify the effect of social deprivation and other risk factors on preterm delivery in high and low risk pregnancies.

### Design, setting and data source

We undertook a retrospective cohort study using routinely collected data from the Liverpool Women's NHS Foundation Trust (LWH). This is the single largest maternity unit in the UK, delivering around 8,000 babies and caring for around 1,000 preterm infants in the neonatal unit annually. We accessed data from the LWH Meditech hospital information system on all women delivering after 24^+0^ weeks gestation over a seven year period from 2002–2008. In order to avoid clustering of risk factors, subsequent pregnancies of women who had more than one pregnancy during the data collection period were excluded from the analysis. The data extracted contained detailed information on demographics, previous and current obstetric history and details of medical conditions.

### Risk stratification

We excluded intrauterine transfers (IUTs), multiple pregnancies, and pregnancies in women with a previous history of preterm delivery <34 weeks from this analysis. The remaining pregnancies were allocated to the high-risk group if they had significant medical conditions. Two obstetricians reviewed all the coded data on co-morbidity in pregnancy, and identified all medical disorders of potential relevance to preterm birth. These included problems identified at booking (e.g. cardiac disease, essential hypertension, epilepsy, diabetes, renal disease, SLE, thyroid disease, Crohn's disease, uterine abnormalities) and problems developed during pregnancy (e.g. gestational hypertension, pre-eclampsia/eclampsia, cholestasis, second trimester vaginal bleeding and Rhesus disease). The group remaining was our low risk population of interest, and represents uncomplicated singleton pregnancies with no identifiable major clinical risk factors for preterm birth.

### Primary outcome and covariates

The primary outcome was preterm birth before 34^+0^ gestational weeks (<238 days: PTB<34) calculated on the basis of first trimester scan. We included both spontaneous and obstetrically induced births less than 34 weeks gestation in this outcome as this group is likely to have significant morbidity, both short and long term, with important resource implications for health services [7], [16]. Preterm birth between 34^+1^ and 36^+6^ weeks (PTB 34–37) was also considered, because the public health burden of late preterm birth is substantial [16]. We hypothesised that the aetiology of late preterm birth may differ from PTB<34.

We aimed to explore the following covariates: maternal age, parity (nulliparous or not), smoking status (never, previous, current smoker<10 cigarrettes per day (cpd), and current smoker>10 cpd, and ), BMI at booking (<18.5 underweight, 18.5 to <35 reference, >35 obese) and ethnicity (self-reported categories coded to white and other). BMI was consistently collected from 2004 onwards in MEDITECH. Before 2004. BMI was calculated from height and weight where available. Postcodes were used to derive Index of Multiple Deprivation (IMD) scores for all of the pregnancies. The IMD combines a number of indicators, chosen to cover a range of economic, social and housing issues, into a single deprivation score for small areas in the UK [17]. Indices of Deprivation 2007 are available for 32,482 small geographical areas (lower super output areas, LSOAs) in England, each containing around 1500 individuals. All of these LSOAs were ranked, and then divided into fifths, providing cut off points for normative English deprivation quintiles (e.g. <8.32, <13.74, <21.22, <34.42, <85.46). Each woman was allocated to one of these quintiles on the basis of IMD score.

### Statistical Methods

Although IMD is measured on a continuous scale, for descriptive summaries, we have followed the common practice of grouping IMD into quintiles. However, reducing IMD to a categorical variable looses information. For formal analysis of the association between deprivation and pre-term birth we therefore retained IMD as a continuous variable. GENREF18 eneralized additive models (GAMs) were used to explore the univariate relationship between the log-odds of preterm birth and deprivation score [18]. The GAMs gave no evidence of a significant non-linear relationship. A logistic regression was therefore used to model parametrically the unadjusted and adjusted relationship between preterm birth, deprivation score and other covariates. The fitted log-odds ratios for IMD score were then used to calculate the OR of preterm birth for a woman at the mid-point of the most deprived English quintile and compared to the mid-point of the least deprived quintile. The potentially mediating role of covariates was explored by comparing the estimates of the association between IMD score and preterm birth before and after including the relevant covariates in the regression model [9] [19]. No a priori sample size calculation was undertaken. Data were complete on all covariates in analysis, other than BMI (15% missing data), which was treated as missing at random. Statistical analysis was undertaken using R (version 2.9.2).

## Results

51,857 pregnancies were recorded in the Meditech system. We excluded 431 intrauterine transfers, 940 multiple pregnancies and 732 pregnancies in women with a history of previous preterm birth, leaving 39,404 low risk pregnancies, and 10,351 high risk pregnancies. Selecting the first pregnancy for each woman during the data collection period resulted in the final sample of 31,785 low risk and 8,130 high-risk pregnancies. A valid postcode was available on all but 42 women leaving 39,873 pregnancies in the final analysis.

**Figure 1:**
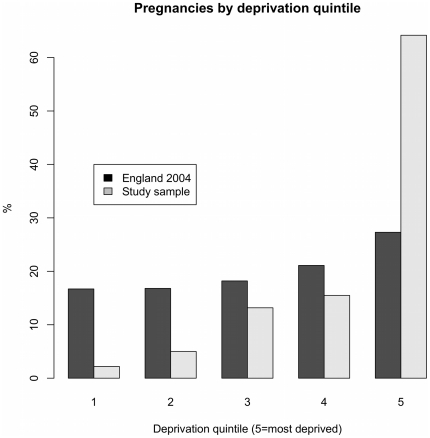
Pregnancies by deprivation quintile for LWH study sample 2002–2008 and all pregnancies in England 2004.

Figure 1 illustrates that the study population was more deprived than the rest of the country, using CEMACH data from 2004 for England as a comparator [20]. In our cohort, 63% of pregnant women came from the most deprived quintile, compared with 27.3% for England in 2004. By contrast, only 2.7% of the LWH sample came from the least deprived quintile, compared with 16.7% for England as a whole. The distribution of deprivation scores were similar in both the high (median = 46.4 and mean = 44.4) and low risk groups (median = 47.4 and mean = 45.1).

Overall, 2.6% (n = 1,036) of pregnancies resulted in preterm birth before 34 weeks, significantly more than 1.9% nationally in 2005 [21]; 572 (55.2%) of these were in the high risk group (n = 8,107) and 464 (44.8%) in the low risk group (n = 31,766) which equates to a preterm birth relative risk of 4.8 (CI_95_ 4.3 to 5.4) comparing the high risk group to the low risk group. Out of these preterm live births there were 53 neonatal deaths before hospital discharge (5.1%) with no difference in the proportion of deaths within the preterm birth subgroups.

There were 1148 (3.6%) late preterm births in the low risk group, and 871 (10.7%) in the high risk group, equating to a late preterm birth relative risk of 2.97 (CI_95_ 2.7 to 3.2) comparing the high risk group to the low risk group.

Table 1 describes the prevalence of potential risk factors for preterm births, and key outcomes in the high and low risk pregnancies, stratified by English normative deprivation quintile. In both groups women from deprived areas were likely to be younger, non-white, either underweight or obese, and smokers. In both groups the proportion of women having a Caesarean section reduced in a graded fashion with increasing deprivation quintile.

**Table 1:** Prevalence of risk factors and outcomes in low and high risk cohorts.

LOW RISK Deprivation quintile	1	2	3	4	5	All	p for trend
N	655	1543	4169	4930	20469	31766	
age<18	1 (0.2)	7 (0.5)	17 (0.4)	36 (0.7)	358 (1.7)	419 (1.3)	0.220
age>30	526 (80.3)	1186 (76.9)	2880 (69.1)	2957 (60)	7871 (38.5)	15420 (48.5)	0.000
White	604 (92.2)	1438 (93.2)	3788 (90.9)	4369 (88.6)	17322 (84.6)	27521 (86.6)	0.000
Underweight	4 (0.7)	20 (1.6)	82 (2.3)	72 (1.7)	600 (3.5)	778 (2.9)	0.000
Obese	61 (11)	149 (11.8)	440 (12.6)	574 (13.8)	2939 (16.9)	4163 (15.5)	0.000
Smoker	37 (5.6)	122 (7.9)	460 (11)	794 (16.1)	7009 (34.2)	8422 (26.5)	0.000
Smoker<10	32 (4.9)	101 (6.6)	380 (9.2)	613 (12.5)	5256 (25.8)	6382 (20.2)	0.000
Smoker>10	5 (0.8)	21 (1.4)	80 (1.9)	181 (3.7)	1753 (8.6)	2040 (6.5)	0.000
Previous smoker	50 (7.7)	139 (9.1)	460 (11.1)	545 (11.1)	2272 (11.2)	3466 (11)	0.005
Nulliparous	350 (53.7)	859 (55.9)	2443 (58.8)	2956 (60.3)	11659 (57.2)	18267 (57.7)	0.817
Caesarean section	162 (24.7)	379 (24.6)	991 (23.8)	1110 (22.5)	3939 (19.2)	6581 (20.7)	0.000
Preterm<34	10 (1.5)	15 (1)	47 (1.1)	61 (1.2)	331 (1.6)	464 (1.5)	0.006
Preterm 34 to 37	17 (2.6)	30 (1.9)	129 (3.1)	157 (3.2)	815 (4)	1148 (3.6)	0.000
Preterm<37	27 (4.1)	45 (2.9)	176 (4.2)	218 (4.4)	1146 (5.6)	1612 (5.1)	0.000
Deaths in preterm<34	0	0	3 (8.5)	5 (8.2)	16 (4.8)	24 (5.2)	0.836

HIGH RISK Deprivation quintile	1	2	3	4	5	All	p for trend
N	216	449	1082	1242	5118	8107	
age<18	3 (1.4)	0 (0)	5 (0.5)	7 (0.6)	48 (0.9)	63 (0.8)	0.082
age>30	172 (79.6)	364 (81.1)	809 (74.8)	843 (67.9)	2369 (46.3)	4557 (56.2)	0.000
White	201 (93.1)	420 (93.5)	1004 (92.8)	1109 (89.3)	4489 (87.7)	7223 (89.1)	0.000
Underweight	1 (0.6)	5 (1.4)	27 (3)	24 (2.3)	171 (4)	228 (3.4)	0.000
Obese	38 (22.4)	54 (15.3)	183 (20.4)	245 (23.3)	992 (23)	1512 (22.3)	0.001
Smoker	12 (5.6)	36 (8)	124 (11.5)	220 (17.7)	1778 (34.7)	2170 (26.8)	0.000
Smoker<10	8 (3.7)	32 (7.3)	100 (9.3)	166 (13.5)	1253 (24.6)	1559 (19.4)	0.000
Smoker>10	4 (1.9)	4 (0.9)	24 (2.2)	54 (4.4)	525 (10.3)	611 (7.6)	0.000
Previous smoker	16 (7.5)	33 (7.5)	93 (8.7)	116 (9.4)	526 (10.3)	784 (9.7)	0.006
Nulliparous	109 (50.7)	237 (53.3)	607 (56.4)	685 (55.5)	2607 (51.1)	4245 (52.6)	0.028
Caesarean section	85 (39.4)	168 (37.4)	370 (34.2)	411 (33.1)	1442 (28.2)	2476 (30.5)	0.000
Preterm<34	22 (10.2)	35 (7.8)	67 (6.2)	73 (5.9)	375 (7.3)	572 (7.1)	0.881
Preterm 34 to 37	21 (9.7)	51 (11.4)	115 (10.6)	107 (8.6)	577 (11.3)	871 (10.7)	0.320
Preterm<37	43 (19.9)	86 (19.2)	182 (16.8)	180 (14.5)	952 (18.6)	1443 (17.8)	0.481
Deaths in preterm<34	2 (9.1)	3 (8.6)	3 (4.5)	6 (8.2)	15 (4)	29 (5)	0.141

**Figure 2:**
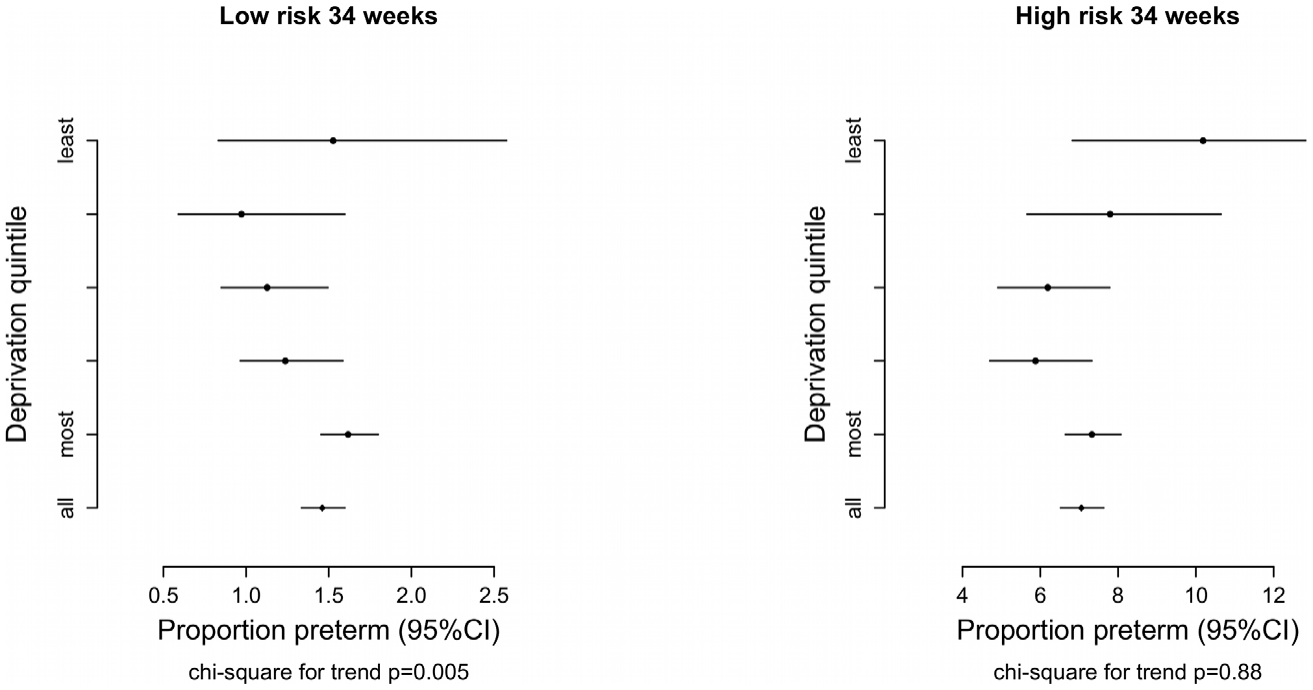
Proportion of PTB in low and high risk groups stratified by deprivation quintile.

**Figure 3:**
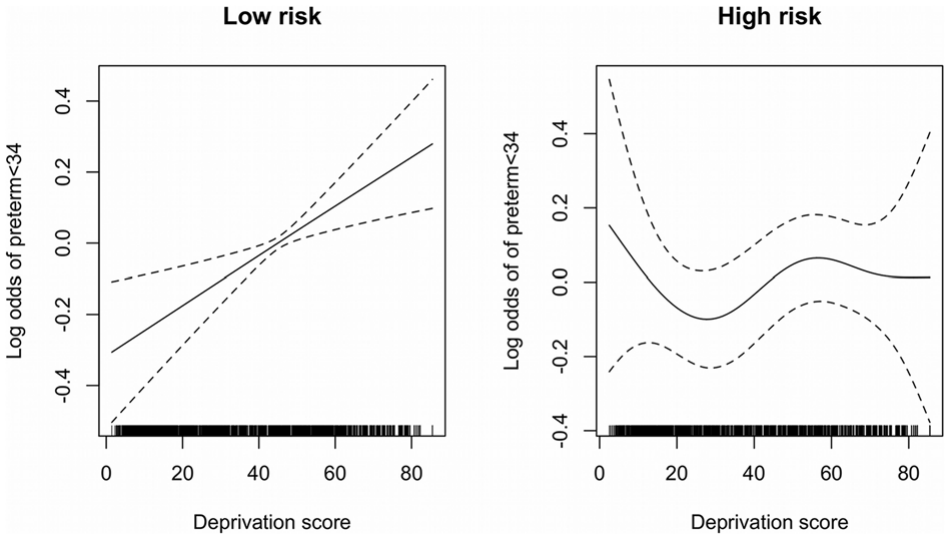
Generalized additive model assessing the relationship between risk of preterm delivery<34 and deprivation score.

Figure 2 shows the proportion of preterm births in the low and high risk groups by deprivation quintile. In the low risk group the preterm proportion was highest in the most deprived quintile (1.6% CI_95_ 1.4 to 1.8) with a significant trend towards a higher proportion of preterm births as deprivation increases (Chi square for trend p<0.01). By contrast, there was no significant trend in preterm births in the high risk group by deprivation quintile. The numbers of deaths was small in both groups with no significant trend detected when these deaths were stratified by deprivation quintile. Figure 3 shows the relationship between deprivation score and the risk of PTB<34 in a generalized additive model (GAM). The relationship is linear on the log-odds scale in the low risk group, and non-significant in the high risk group.

Table 2 illustrates the univariate association of covariates of interest on the risk of preterm birth<34 in the low and high risk groups, with IMD score treated as a continuous variable in logistic regression. In the low risk group, the unadjusted OR for preterm birth was 1.0069 per unit increase in IMD (CI_95_ 1.003 to 1.0114), which equates to an OR of 1.47 (CI_95_ 1.16 to 1.86) comparing an individual in the most deprived quintile to one in the least deprived quintile (ie. 1.0069 raised to the power 55, which is the difference between the midpoint of quintile 1 and 5). Age<18, being underweight and smoking were also highly significantly associated with an increased risk of preterm birth.

**Table 2:** Univariate regression assessing association between covariates and PTB in low and high risk group.

LOW RISK								
	PTB 34				PTB 34–37			
	OR	2.50%	97.50%	p	OR	2.50%	97.50%	p
Most deprived	1.47	1.16	1.86	0.00	1.56	1.34	1.82	0.00
age<18	1.97	1.04	3.38	0.02	1.12	0.66	1.77	0.66
age>35	0.91	0.73	1.14	0.44	0.96	0.83	1.11	0.61
Underweight	2.34	1.47	3.55	0.00	1.43	1.01	1.96	0.03
Obese	0.90	0.65	1.22	0.52	0.97	0.81	1.16	0.75
smoker<10	1.45	1.16	1.80	0.00	1.57	1.37	1.81	0.00
smoker>10	1.44	1.01	2.01	0.04	2.00	1.63	2.44	0.00
smoker previous	0.89	0.62	1.22	0.48	1.05	0.85	1.28	0.64
white	0.97	0.75	1.27	0.80	1.17	0.98	1.41	0.10

HIGH RISK								
	PTB 34				PTB 34–37			
	OR	2.50%	97.50%	p	OR	2.50%	97.50%	p
Most deprived	1.07	0.86	1.33	0.53	1.26	1.05	1.51	0.01
age<18	0.88	0.27	2.16	0.81	0.90	0.34	1.92	0.80
age>35	1.07	0.89	1.28	0.47	1.08	0.93	1.26	0.31
Underweight	1.28	0.75	2.04	0.33	1.69	1.16	2.40	0.00
Obese	0.84	0.65	1.08	0.18	1.03	0.85	1.24	0.78
smoker<10	0.85	0.67	1.06	0.15	1.40	1.17	1.66	0.00
smoker>10	1.27	0.94	1.68	0.11	1.64	1.29	2.08	0.00
smoker previous	0.64	0.45	0.89	0.01	0.97	0.74	1.24	0.79
white	0.94	0.72	1.23	0.62	0.78	0.63	0.96	0.02

For low risk late preterm births there was a similar relationship with deprivation, smoking and being underweight, but age<18 was not significant. For high risk late preterm births the most deprived quintile had an increased risk of preterm birth (OR 1.26 CI_95_ 1.05 to 1.51), as did smokers, being underweight, and non-white women.

Table 3 explores the effect of deprivation of preterm birth, adjusted for BMI and smoking. In the low risk women, the adjusted OR for PTB<34, comparing the most to the least deprived quintile, lost the significance at 5% level (1.25 CI_95_ 0.90 to 1.73). Smoking and being underweight remained highly significant, both approximately doubling the risk of preterm birth<34. For late preterm births smoking had a similar effect, but underweight was not significant, and the adjusted OR for the effect of deprivation was reduced, but remained significant (1.32 CI_95_ 1.12 to 1.64). In high risk women, smoking was associated with both preterm subgroups, but being underweight was only significant for preterm births between 34–37 weeks.

**Table 3:** Multivariate logistic regression models assessing independent association between covariates and PTB in low risk group.

LOW RISK	PTB34				PTB34 to 37			
	OR	2.50%	97.50%	p	OR	2.50%	97.50%	p
most deprived unadjusted	1.47	1.16	1.86	0.00	1.56	1.34	1.82	0.00
most deprived adjusted	1.25	0.90	1.73	0.16	1.32	1.12	1.64	0.00
smoker<10	1.82	1.40	2.36	0.00	1.50	1.28	1.76	0.00
smoker>10	1.93	1.30	2.78	0.00	1.79	1.42	2.23	0.00
smoker - previous	1.15	0.79	1.65	0.44	1.03	0.83	1.28	0.76
Underweight	2.11	1.31	3.21	0.00	1.28	0.90	1.77	0.15
Obese	0.91	0.65	1.23	0.55	0.95	0.79	1.14	0.61
age<18	1.82	0.86	3.38	0.08	0.87	0.46	1.49	0.63
age>35	1.11	0.84	1.45	0.45	1.06	0.90	1.25	0.47

HIGH RISK	PTB34				PTB34 to 37			
	OR	2.50%	97.50%	p	OR	2.50%	97.50%	p
most deprived unadjusted	1.07	0.86	1.33	0.53	1.26	1.05	1.51	0.01
most deprived adjusted	1.23	0.93	1.61	0.14	1.00	0.83	1.28	0.73
smoker<10	0.98	0.75	1.27	0.88	1.37	1.12	1.67	0.00
smoker>10	1.55	1.10	2.14	0.01	1.84	1.41	2.39	0.00
smoker - previous	0.86	0.59	1.21	0.40	1.02	0.77	1.34	0.86
Underweight	1.27	0.74	2.03	0.35	1.64	1.12	2.34	0.01
Obese	0.84	0.65	1.08	0.19	1.06	0.88	1.28	0.53
age<18	0.29	0.02	1.32	0.22	0.85	0.29	1.96	0.74
age>35	1.02	0.81	1.28	0.84	1.16	0.97	1.38	0.10

## Discussion

Using routinely collected obstetric data from a retrospective cohort of 39,873 women with a singleton pregnancy, we were able to define a high risk group with a five fold greater risk of preterm birth before 34 weeks compared with the women without obvious risk factors. In otherwise low risk pregnant women deprivation, age <18, underweight and smoking were associated with preterm birth<34.

Our data, therefore, suggest that reducing the burden of disease due to very preterm birth will have to involve targeted, disease specific preventative interventions in high risk pregnancies, but for low risk groups, population level public health action is needed to address risk factors for preterm birth associated with social deprivation.

In women who gave birth between 34 and 37 weeks the distinction between low and high groups was less distinct – smoking was important, but age and obesity were not. Interestingly, being underweight was significant in high risk, but not in low risk. The opposite was found in very preterm births. The reasons for this pattern are unclear. Despite the large number of pregnancies analyzed, it is likely that our analysis was underpowered to detect a statistically significant association with being underweight in some of our subgroups. All of the point estimates for underweight are in the same direction, however, suggesting that underweight women are at increased risk of preterm birth.

We have demonstrated that it is possible to identify distinct clinically important groups, with markedly different preterm birth rates, on the basis of medical information collected during pregnancy. In-utero transfers, multiple pregnancies and women with a history of previous preterm birth have very different risks of preterm birth (results not reported here) and should be analysed separately. We suggest that stratification of birth outcomes in terms of these groups provides a more meaningful method to report preterm birth outcomes from maternity units for auditing and benchmarking purposes. So for example, rates of preterm birth in multiple pregnancies would provide an additional useful metric.

Reducing infant mortality, which is accounted for to a great extent by preterm birth, is a key focus of both the recent NHS white paper, and the Public Health white paper [22], [23]. ENREF19 Furthermore, addressing inequalities in infant mortality rate has been a political imperative in the UK over the last 10 years. The UK government's latest report on health inequalities suggests that one quarter of infant deaths would potentially be avoided if all births had the same level of risk as those to women with the lowest level of deprivation [1].

It has been hypothesised that the relationship between low socioeconomic status and preterm birth may be explained by the clustering of demographic and ‘lifestyle’ risk factors in women from more disadvantaged backgrounds [6], [8], [24]. This is supported by our analysis. There are striking social gradients evident in these risk factors, which appear to be mediating some of the effect of social deprivation: In our low risk population adjusting for smoking and low maternal weight removed the significant association between SES on PTB<34, and reduced the odds ratio for late preterm birth from 1.56 to 1.32. This is in line with a number of other studies in different settings. A population wide Danish study found the educational gradient in risk of preterm birth was reduced after adjustment for factors including smoking, BMI and alcohol consumption [9]. In a multi-level US study, however, a consistent effect of area level deprivation was found even after accounting for individual demographic, obstetric, behavioural, and medical risk factors [8]. In a Scottish study smoking status at first antenatal contact and increased obstetric intervention appeared to explain some, but not all the social gradient in outcomes [25].

In our cohort, being underweight (BMI<18.5) emerged as a clear risk factor in the low risk group, approximately doubling the risk of preterm birth<34. This is in line with a recent meta-analysis [21] and though underweight women account for a small proportion of all pregnancies (around 3% in our population) they are a potential target for intervention. It has been postulated that decreased blood volume, reduced uterine blood flow and low concentration of vitamins and minerals leading to maternal infections may be implicated [7]. On the other hand, we found no association between obesity and preterm births. Previous studies have found an inconsistent relationship between high BMI and the risk of preterm birth, with a recent meta-analysis concluding that “high maternal BMI may have different effects on different types of preterm births.” [26].

A strength of our analysis is that we have a large sample, with individual level clinical data, and were able to stratify pregnancies in terms of major obstetric risk factors. The main purpose of this stratification was to allow a ‘cleaner’ view of the relationship between SES and preterm births, without the risk of confounding, particularly by medical problems. For this reason, we stratified the pregnancies based on a combination of characteristics that may or may not be present in early pregnancy. A different analytical approach would be required to quantify the risk of preterm birth for counselling purposes in early pregnancy. These methods and our findings are likely to be generalizable to other large tertiary maternity units in the UK, serving deprived populations.

### Limitations

This study is hospital based, rather than population based, but our cohort is likely to be similar to a population based cohort, since LWH is the main maternity unit in the Liverpool area, and there are no private providers. We have limited selection bias by excluding transfers in to LWH, who have a different risk profile. We had to rely on retrospective, routinely collected data, and there is scope for response bias in the self-reported smoking status covariate. We have used a standard small area based measure of deprivation. It was therefore not possible to separate effects of SES operating at the individual and area level, which may be distinct, as suggested in other studies of preterm births [8], [9], [10], [27], [28].

### Conclusions

Our results have a number of implications. Social deprivation is an important risk factor for preterm birth, and the effect of deprivation is related to maternal smoking and underweight, providing clear targets for public health action to reduce inequalities in preterm birth and subsequent infant mortality. At the individual level smoking is recognised as one of the most important “lifestyle” factors in the pathway to health inequalities, and persistent social gradients remain in the UK [29]. Intensification of efforts to ensure that women stop smoking before becoming pregnant is a priority.

The social distribution of preterm birth suggests that social factors – the “social determinants of health” – are having an important effect on outcomes. These are the “conditions in which we are born, grow up, work and live” [1], and include things such as a decent education, adequate housing, being able to access a nutritious diet, and having the financial resources to engage fully in society. It is likely that the socioeconomic differences in preterm birth cannot be adequately remedied at the individual level, and that individually focussed interventions need to be complemented by broader action to address the social inequalities that influence health over the course of people's lives. Suggested approaches are outlined in the recent UK Marmot report, the key recommendation of which is “to give every child the best start in life”, advocating more social investment in the antenatal period and early years [1].

Further attempts to explore the pathways from low SES to preterm births need to take into account individual and area level mediators to identify further targets for preventative interventions. As a way forward, maternity units should produce preterm birth rates stratified by clinical risk factors and deprivation quintiles. Given the influence of SES, the proportion of preterm and term births to women in the most deprived normative English quintile should be used as a metric in comparing quality of maternity care across centers. Targeted analysis of outliers will provide important insights into possible preventative interventions and resource allocation by newly established GP consortia in the UK [23].
